# Experiences of Older Mental Health Patients and Their Care Partners Using a Proxy Account to Access Open Notes: Qualitative Interview Study

**DOI:** 10.2196/66690

**Published:** 2025-02-24

**Authors:** Eva Meier-Diedrich, Tobias Esch, Maria Hägglund, Martin Heinze, Stefan Hochwarter, Justin Speck, Marie Wagener, Volker Dahling, Julian Schwarz

**Affiliations:** 1 Department of Psychiatry and Psychotherapy, Center for Mental Health Immanuel Hospital Rüdersdorf Brandenburg Medical School Theodor Fontane Rüdersdorf Germany; 2 Faculty of Health Sciences Brandenburg Brandenburg Medical School Theodor Fontane Neuruppin Germany; 3 Institute for Integrative Health Care and Health Promotion, Faculty of Health School of Medicine Witten/Herdecke University Witten Germany; 4 Department of Women’s and Children’s Health Uppsala University Uppsala Sweden; 5 Uppsala University Hospital Uppsala Sweden; 6 Center for Health Service Research Brandenburg Brandenburg Medical School Theodor Fontane Rüdersdorf Germany; 7 Institute for Biomedicine and Health Sciences (HEALTH) Joanneum Research Forschungsgesellschaft m.b.H Graz Austria; 8 Research Group Geriatric Psychiatry Research Brandenburg Medical School Theodor Fontane Rüdersdorf Germany

**Keywords:** psychiatry, eHealth, mental health, digital literacy, older patients, older adult, care partner, proxy access, open record access, Open Notes, patient portal, artificial intelligence, AI

## Abstract

**Background:**

Older patients with serious mental illnesses such as cognitive disorders often rely on family members or spouses (care partners [CPs]) to meet their health care needs. CPs frequently lack essential information to fully understand the patients’ illnesses and effectively support their treatment. Open Notes provide patients with digital access to their health care professionals’ clinical notes and are associated with many positive outcomes, such as increased adherence and empowerment. However, older patients who use Open Notes may encounter use barriers such as limited digital literacy. Recent developments allow CPs to access Open Notes (proxy access) and receive valuable information, which holds significant potential for improving the care of older patients.

**Objective:**

This study explored the experiences, barriers, and opportunities of older mental health patients and their CPs related to using Open Notes. Furthermore, influencing factors and interdependencies were identified.

**Methods:**

Older patients (n=10) and their CPs (n=10) were provided with web-based proxy access to clinical documentation through a web-based patient portal. In-depth qualitative interviews (N=20) were conducted to explore experiences with this access. Data analysis was conducted in accordance with the constructivist grounded theory approach.

**Results:**

The prerequisites for using Open Notes with proxy access were sufficient digital literacy on the part of the patient or CP, as well as the establishment of a trusting relationship between patients and CPs. Access to Open Notes enabled patients and CPs to gain a deeper understanding of the illness and its treatment while also facilitating enhanced contact with health care professionals. This resulted in greater involvement in the treatment process but may also prompt changes in relationship dynamics—CPs are better equipped to support patients in their health care but may also tend to monitor or control them through Open Notes. As a result, the introduction of Open Notes was accompanied by mixed feelings.

**Conclusions:**

It is of utmost importance to provide older patients with comprehensive access to Open Notes to preserve their health autonomy. However, the involvement of CPs through proxy access is of great value in improving the care of older patients, especially those with cognitive impairments.

## Introduction

### Background

The ongoing digitalization throughout society, coupled with demographic transformations, presents both opportunities and challenges for the health care sector. The global population is living longer, and in particular in industrialized countries, fertility rates are declining, which is significantly altering the age composition of the population [1]. Concurrently, digitalization is gradually permeating all areas of society, including the mental health care sector, thereby providing patients with new opportunities to participate in their treatments [2]. The European Health Data Space proposal follows this development and aims to improve patients’ access and control over their personal electronic health data [3]. One such opportunity for patient participation is through online record access (ORA), which enables patients to view their electronic health records (EHRs) via web-based patient portals. When patients are able to also access their health care professionals’ (HCPs) notes via ORA, this is referred to as Open Notes [4-6]. In the United States and some Scandinavian countries, Open Notes are already an established practice in health care [3,7]. Patients in the United Kingdom have the option of accessing the clinical notes of their general practitioners [8]. In contrast, in other countries such as Germany, Open Notes have yet to be integrated into the health care system [9,10]. However, current legislation requires all hospitals in Germany to implement patient portals. Therefore, in the near future, it will be technically possible to offer Open Notes [11]. Access to clinical notes and the associated transparency can enhance patient empowerment and engagement, improve medication adherence, and bolster disease management and awareness [12-15]. Studies indicate that vulnerable groups, including older patients, particularly benefit from Open Notes [16]. Furthermore, it can enhance communication between (older) patients and their HCPs, thereby strengthening trust in the treatment process [17,18]. While many older patients express interest in and intent to use digital health technologies, few actually use them [19]. Because older patients are not considered digital natives, they often face several challenges when using digital health technologies, including limited digital health literacy, usability issues, and heightened concerns about data security [19-21]. In addition, older patients are increasingly affected by cognitive deficits (eg, dementia), which further complicates the development of digital health literacy [22]. These barriers lead to a decrease in the use of web-based health services such as ORA with advancing age, especially without (human) guidance [23]. Therefore, it is imperative that digital health literacy is taken into account in the design of patient portals and EHRs to ensure their accessibility and inclusivity for older users [24,25].

In general, a significant proportion of older patients rely on relatives such as family members, partners, or friends (referred to as care partners [CPs]) to meet their health care needs [24]. This is also true for digital health services [26]. Receiving support from CPs has a positive impact on patients’ quality of life, quality of care, and health resource use. In addition, patients and CPs would like to have greater involvement in medical care, but this has not been adequately supported by the health care system [24,27]. CPs often lack essential information about the health status and treatment planning of older patients, which can significantly complicate care [24]. With proxy access, Open Notes allow relatives to read medical treatment documentation with the patient’s consent [28]. Giving CPs access to Open Notes provides them with important information and facilitates their care. In addition, studies show that the involvement of CPs can also increase engagement in the treatment of (older) patients using ORA [29]. Current research confirms that such access is desired by CPs [22,30] but acceptance is still limited [26]. ORA and Open Notes will become increasingly important in health care, and the associated opportunities should be available to all patients [22,24]. Therefore, it is essential to gain a deeper understanding of the use patterns of older patients, the role of CPs, and the barriers and opportunities associated with them.

### Objectives

To date, only a limited number of studies have examined the use of ORA by older patients in the context of proxy access [24,31,32]. Specifically, in the area of mental health, the authors are currently unaware of any studies on this topic [33,34]. In light of the current state of research, the purpose of this study was to explore the experiences, preferences, and needs of older mental health patients and their CPs, as well as the barriers and opportunities related to using Open Notes. In addition, this study aimed to provide recommendations for best practice in this area and sought to identify the factors influencing the impact of CP access to Open Notes.

## Methods

### Study Design

The *Piloting and evaluation of a participatory patient-accessible electronic health record for geriatric psychiatric patients and their care partners* (PEP.AGE) study is part of the *Piloting and evaluation of a participatory patient-accessible electronic health record in Psychiatry and Somatics* (PEPPPSY) project (2021-2026) [35,36]. In the PEPPPSY project, patients are provided with access to their HCPs’ treatment and progress notes via a dedicated patient portal [37]. Furthermore, the development and implementation of the patient portal are being examined from the dual perspectives of both patients and HCPs. The PEP.AGE study broadens the scope of the PEPPPSY target population by including not only the perspective of older patients but also that of their CPs. Given the exploratory nature of this study, a qualitative design was chosen to ensure a comprehensive and thorough examination of the use of Open Notes by older patients and their CPs.

### Ethical Considerations

This study was approved by the ethics committee of Brandenburg Medical School Theodor Fontane (E-01-20210727) and registered with the German Clinical Trials Register (DRKS00030188). Participants were informed of the study content and procedures both verbally and in writing. Informed written consent was then obtained from all participants. All participants had the right to withdraw from the study at any time without any adverse consequences. All data were anonymized. Participants received a compensation of €40 (US $41.39) for their participation.

### PEPPPSY App

The patient portal pilot, called PEPPPSY, was initiated as part of a research collaboration between the Norwegian University of Science and Technology and Brandenburg Medical School Theodor Fontane. The portal was developed through an ongoing iterative and participatory process [35,36]. In addition to accessing clinical notes, patients and CPs can respond to HCPs’ entries with comments. HCPs are then notified of these comments and can respond to patients (or CPs) within the same thread. In the current second phase of the project, the pilot has been expanded to include access for CPs (proxy access), which will increase the accessibility and utility of the portal for a broader patient population.

### Study Setting

This study was conducted in 2 psychiatric outpatient clinics (Rüdersdorf and Strausberg) of the Immanuel Hospital Rüdersdorf in the state of Brandenburg, Germany. Psychiatric outpatient clinics are specialized facilities that provide psychiatric care to patients with severe mental illness. These patients typically require comprehensive and multidisciplinary psychiatric treatment and often lack access to adequate care in other outpatient settings (such as psychiatric or general medical practices) due to the severity or chronicity of their psychiatric conditions.

### Recruitment

Eligible participants were enrolled in the PEP.AGE study from June 2023 to January 2024. Participating patients had to be aged ≥60 years, receive treatment at 1 of the 2 designated sites, and be able to provide informed consent. Patients with risk factors such as self-harm or harm to others and severe cognitive impairment were excluded from the study. Participating CPs had to be adults (aged ≥18 years) and able to provide informed consent.

### Data Collection

At enrollment, sociodemographic data were collected from both patients and their CPs. Patients and CPs were then introduced to the use of the patient portal by their HCP or a member of the study team. They were provided with a comprehensive, user-friendly manual and the option of one-on-one assistance to set up their accounts and learn how to use Open Notes step by step. Subsequently, the older patients and their CPs (with patient consent) were given access to the patient portal and the HCPs’ clinical notes. At the beginning of the intervention phase, participants were randomly contacted to identify and address any barriers to use. At the end of the 3-month intervention phase, semistructured interviews were conducted with patients and CPs using previously developed interview guides to gain deeper insights into their actual experiences with Open Notes (Multimedia Appendix 1). The interviews lasted between 20 and 30 minutes each. Throughout the study (onboarding phase, intervention phase, and interview phase), the study team kept field notes documenting observations and contextual information [38].

### Data Analysis

The qualitative interviews were audio recorded, pseudonymized, transcribed, and analyzed by 2 researchers with the computer-assisted analysis software MAXQDA (VERBI GmbH) using the constructivist grounded theory by Charmaz [39]. The selected analytical approach was appropriate to the research subject as this exploratory study aimed to iteratively develop theoretical concepts from the data. In accordance with the approach by Charmaz [39], the data analysis was conducted continuously, commencing with the earliest data gathering (initial interview). The interviews were initially coded line by line to facilitate the conceptualization of ideas and the development of preliminary codes. Subsequently, focused coding was conducted, whereby the most significant and frequent codes were identified, sorted, and synthesized into overarching categories. Following this, relationships between the categories were identified and connected into coherent theoretical concepts (theoretical coding). On the basis of the developed concepts and emerging theory, the research team returned to the field and gathered additional data on specific themes until theoretical saturation was achieved. For quality assurance purposes, the COREQ (Consolidated Criteria for Reporting Qualitative Research) checklist was used (Multimedia Appendix 2).

## Results

### Sociodemographic Data

A total of 10 patients and 10 CPs were interviewed via telephone (by EMD or MW), and their complete sociodemographic data are shown in Table 1. In total, 5 dyads (each consisting of a patient and their respective CP), as well as 5 independent patients and 5 independent CPs, were interviewed. All participants self-identified as White individuals, were born in Germany, and spoke German as their native language. The age of the patients ranged from 62 to 81 years, with a mean of 71.60 (SD 6.43) years. A total of 70% (7/10) of the patients identified as female, and 30% (3/10) identified as male. Most patients (8/10, 80%) were retired, whereas a minority (2/10, 20%) were still employed. All CPs (10/10, 100%) were family members of the patients (mainly spouses or children). The ages of the CPs were more diverse, ranging from 45 to 81 (mean 61.20, SD 11.02) years. This was due to the participation of spouses (5/10, 50%), children (4/10, 40%), and other family members (1/10, 10%) of the patients as CPs. In total, 40% (4/10) of the CPs were already retired, whereas 60% (6/10) were still employed part time or full time. Before the start of the PEP.AGE study, the vast majority (8/10, 80%) of the older patients had already given their relatives access to their health information. Patients reported medical discussions with their HCPs to their CPs, shared medical correspondence and medication schedules with them, or were accompanied by CPs to medical appointments.

**Table 1:** Characteristics of the patient–care partner dyads.

Dyad number	Patients				Care partners		
	Age (y)	Sex	Diagnosis (*ICD-10*^a^ code)	Age (y)		Relationship to patient	Employment status
1	67	Female	Mild cognitive disorder (F06.7)	62		Spouse	Part time
2	75	Female	Dementia in Alzheimer disease with late onset (F00.1)	51		Child	Full time
3	80	Male	Dementia in Alzheimer disease with late onset (F00.1)	69		Spouse	Retired
4	81	Female	Severe depressive episode without psychotic symptoms (F32.2)	45		Child	Full time
5	67	Female	Social phobias (F40.1)	69		Spouse	Retired
6^b^	69	Female	Recurrent depressive disorder, current episode severe without psychotic symptoms (F33.2)	52		Child	Retired
7^b^	62	Male	Generalized anxiety disorder (F41.1)	53		Child	Full time
8^b^	68	Male	Recurrent depressive disorder, current episode severe without psychotic symptoms (F33.2)	81		Spouse	Retired
9^b^	78	Female	Bipolar affective disorder, current episode mild or moderate depression (F31.3)	69		Spouse	Retired
10^b^	69	Female	Recurrent depressive disorder, current episode severe without psychotic symptoms (F33.2)	61		Other family member	Full time

^a^ICD-10: International Classification of Diseases, 10th Revision.

^b^No dyad; independent care partners and patients.

The patients supported by the independent CPs (CPs 6-10) were aged between 71 and 86 (mean 80.8, SD 6.099) years and retired, and they self-identified as White individuals. Patients were being treated for the following main diagnoses: dementia in Alzheimer disease with late onset (*International Classification of Diseases, 10th Revision* [*ICD-10*], code F00.1); mild cognitive disorder (*ICD-10* code F06.7); recurrent depressive disorder, current episode severe without psychotic symptoms (*ICD-10* code F33.2); and mixed anxiety and depressive disorder (*ICD-10* code F41.2).

### Qualitative Findings

#### Overview

**Figure 1:**
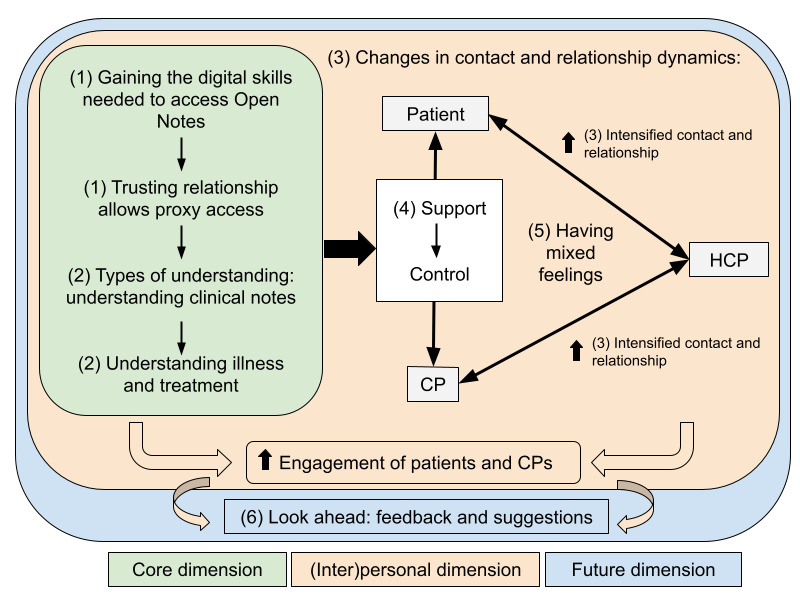
Interrelations of the qualitative categories and dimensions related to the use of Open Notes with proxy access by older patients and their care partners (CPs). Arrows pointing upward indicate increase or enhancement. HCP: health care professional.

The results revealed 3 (partially interrelated) dimensions associated with Open Notes with proxy access when used by older patients and their CPs. These dimensions and their interactions are summarized in Figure 1 and described in detail in the following sections. The green core dimension in Figure 1 provides the foundation for the use of Open Notes and proxy access (eg, digital skills and literacy, trust, and understanding of note content). The red (inter)personal dimension encompasses the impact that Open Notes can have on the relationships among patients, CPs, and HCPs. Finally, the blue future dimension offers ideas and recommendations for the further development of Open Notes. Quotations from CPs and patients are identified using the IDs *CP* and *PAT*, respectively.

#### Gaining the Digital Skills Needed to Access Open Notes

The interviews and field observations revealed that the digital health literacy of the older patients and their CPs varied widely—both between dyads (patient-care-partner pairs) and across participant groups. While some dyads required direct assistance from study team members to activate, log into, and use the patient portal, others required no assistance at all. Younger CPs generally found it much easier to navigate the patient portal than older patients. This variability was also evident in the interviews, with approximately half (4/10, 40%) of the patients and most of the CPs (6/10, 60%) reporting that using Open Notes was challenging or even beyond the digital literacy of the patients, as illustrated by the following statements:

It [technical difficulties] could very well be because I’m no longer able to do things like that. [I had] Two strokes and then the nerve disease.

PAT_0420

New technologies are often a challenge for older people. It takes a lot of patience and support from us younger ones, but it’s doable if you stick with it and show them they can do it.

CP_0213

As the previous quotes illustrate, a significant number of patients (5/10, 50%) relied on the assistance of their CPs to use the patient portal and Open Notes. At the same time, 20% (2/10) of the CPs themselves indicated that using digital health technologies posed a (manageable) challenge to their digital literacy:

I had to overcome a few technical hurdles, but with time and some support, I managed to use the application.

CP_0213

The 2-factor authentication log-in process proved to be particularly challenging, if not insurmountable, for some patients and CPs. Some participants were able to overcome this barrier on their own or with assistance (from CPs or the study team). However, others became so frustrated that they stopped using the patient portal altogether. CPs reported the following:

Well, there’s this two-step login process, where you need the SMS PIN, and then—since I mostly used it on mobile—you have to fiddle around a bit to find where the access to the records is, but ultimately it was okay.

CP_0209

I had a question once because the access didn’t work at the beginning, but it was resolved relatively quickly. They sent me a new one, and I was able to use it. Okay, but I don’t know exactly if it was my fault or if it was just issues with the program.

CP_0409

Although most participants reported using and perceiving benefits from the patient portal, 30% (3/10) of the CPs and 10% (1/10) of the patients indicated that they did not access the patient portal in their daily lives. This was due to a lack of perceived need to review the information given their regular contact with the HCP and the absence of a crisis situation that would have made accessing the records more relevant:

For us, it [Open Notes] doesn’t have any everyday use. The idea that there’s the possibility to look up and comment on disputed questions is great. But in the six months we’ve been participating, there hasn’t been a situation that required us to intervene or do anything.

CP_0211

Most patients (8/10, 80%) agreed that a solid foundation of trust is a prerequisite for disclosing sensitive clinical information to family members. Some CPs (3/10, 30%) expressed a similar view. An open and honest conversation about the advantages and disadvantages regarding the patients’ privacy seemed to be particularly important before using Open Notes with a proxy access, as the following quote shows:

We had discussed beforehand what this is and what it means. Otherwise, I don’t think she [CP] would have agreed to it. When everything is disclosed, you have to be willing to accept that. Some might say, “Oh no, I don’t want that, it’s too private,” depending on who it is and the relationship involved.

PAT_0419

#### Types of Understanding

##### Overview

The implementation of Open Notes involves several types of understanding. First, it is essential for patients and CPs to comprehend the content of the clinical note, which requires that the notes be written in patient-friendly language. In addition, by reading the clinical notes, older patients and their CPs were able to gain a better understanding of the illness and its treatment. This allowed them to adequately prepare for medical appointments, reducing anxiety and facilitating understanding during treatment sessions.

##### Understanding Clinical Notes

Most interviewed patients (8/10, 80%) and half (5/10, 50%) of the CPs agreed that the documentation was particularly understandable when it was composed in a manner accessible to patients without a lot of medical or technical jargon:

It [Open Notes] was understandable. Without any medical jargon, everything was fine. The way I described it, he [the HCP] wrote it down, more or less in my own words. It was expressed a bit better, but still in normal, understandable terms, I would say.

PAT_0411

Yes, it was understandable. Of course, there are always medical terms that might be unfamiliar to a layperson at first. But then you remember the conversation and can figure out what it was about and what was meant. So far, I can’t say that I didn’t understand anything. It was all very understandable.

CP_0201

The previous quotes show that, for some users, the information in the Open Notes alone is not sufficient; rather, understanding is built by combining the knowledge gained from the conversation with HCPs during in-person medical appointments and the information provided in the Open Notes.

Some CPs accompanied patients to their medical appointments and were able to recall the content of the conversations. However, for CPs who were unable to attend appointments, the clinical notes seemed to be easy to understand, as the following quote illustrates:

Yes, those were his notes. Brief and to the point. Of course, he didn’t write long texts, but at least he documented briefly what was discussed, how the medications are, and what the plan is going forward. He wrote it down in a way that was understandable for everyone.

CP_0416

##### Understanding the Illness and Its Treatment

Some patients (3/10, 30%), but especially CPs (6/10, 60%), reported that access to patient-friendly clinical notes provided them with a more complete understanding and awareness of the patient’s illness and treatment:

I now have a much better overview of the entire treatment process and my mother’s current health condition. This makes it easier to make informed decisions and plan the next steps.

CP_210

This improved understanding of the illness appeared to serve as a foundation for subsequent developments, including the increased involvement of CPs in the treatment process and the provision of support. Because treatment appointments often leave little time for questions or repetition, patients (5/10, 50%) and CPs (3/10, 30%) found open-ended notes to be a valuable reminder, allowing them to prepare for and follow up on appointments more easily. Participants found it beneficial to have a written record of what was discussed that they could review at their own pace, allowing them to process the information in a way that best met their individual needs:

Remembering and understanding important details and conversation points better. This was especially useful for preparing for appointments and following up on recommendations.

CP_0213

Because for me, it’s easier when I see something in writing, read it, and then respond or share my own experiences. This back-and-forth, this exchange with the doctors and staff, it’s easier for me in writing than sitting in front of the doctor who might not have much time.

PAT_0402

If we have an appointment, we review the last one together, summarize the key points, and build the new medical appointment on that.

CP_0201

As the preceding quotes illustrate, one patient noted that reviewing the documentation allowed her to better assess and understand her own treatment progress. Another patient reported that he used the clinical notes to confirm that his HCP had understood him correctly during the visit. This gave him the opportunity to address any potential misunderstandings:

Exactly, it allows you to see for yourself that everything was conveyed clearly. When you only attend the doctor’s visit and then leave, you forget half of it anyway. It was much better for me to be able to read it again and confirm that I was truly understood.

PAT_0204

#### Changes in Contact and Relationship Dynamics

Nevertheless, many patients (7/10, 70%) and CPs (5/10, 50%) described Open Notes as facilitating communication with HCPs in a variety of ways. Open Notes, with the opportunity to comment on notes and send messages back and forth, made contact with HCPs faster, more direct, more efficient, more accessible, and more frequent. As a result, participants reported an increased sense of involvement in the treatment process and improved collaboration with HCPs:

You can communicate quickly. That is important. It’s nice, it’s accessible.

CP_0409

Yes, the contact became more frequent, and I felt more involved in the treatment. It was a very positive effect that improved collaboration.

CP_0210

In addition, Open Notes allowed participants to contact HCPs outside of office hours, which was particularly convenient for full-time CPs. All participants were aware that this was asynchronous communication and that responses would only be made during the HCPs’ working hours. One patient said the following:

You can exchange messages, even on the quick...So having a direct line to the doctor [through Open Notes], without having to call during office hours, is relatively quick. You get a prompt response from the doctor to what you write.

PAT_0204

In some cases, communication via Open Notes even replaced telephone contact. This was seen as a relief by some participants (1/10, 10% of patients and 4/10, 40% of CPs) as HCPs were often difficult to reach by phone. In this context, one CP highlighted the portal’s communication and commenting function as a valuable tool for patients with mental health conditions who may find phone calls challenging:

Interviewer: Okay. And compared to calling, did you feel that you could reach your healthcare provider better or faster through the patient portal than by phone or other means?...Patient: Definitely much better, because calling is always tricky. If they’re in treatment, you can’t reach them. But this way, they responded when they had time, and everything was handled very quickly, so it was totally fine.

PAT_0204

As I would say, when patients use it themselves, and I am not a patient, but patients generally have underlying issues, often psychological, which make it difficult for them to communicate. Yes, reaching for the phone is challenging, going to the doctor is difficult. But maybe writing is somewhat less personal and might be easier. And it can be done at night or at an inconvenient time without feeling guilty, so I can imagine this is definitely a good option that could continue to be used.

CP_0409

Moreover, a slight shift in the relationship dynamics between patients and their CPs was noted with the implementation of Open Notes with proxy access. Now that CPs had access to the clinical notes, some patients (4/10, 40%) felt that their CPs were more understanding of their mental illness. This suggests a developmental process on the part of the CPs initiated by reading the shared notes:

Well, my stepson initially had problems because he couldn’t imagine it when I said that it’s still hard for me to take the bus alone. I get such a racing heart...But as I said, my stepson couldn’t really understand it. Maybe you can’t fully understand if you are healthy. But he has learned to understand it.

PAT_0224

#### From Support to Control

There was considerable variability in the level of digital health literacy among the older patients (see the *Gaining the Digital Skills Needed to Access Open Notes* section). In particular, patients in the oldest age group and those with cognitive impairments relied heavily on their CPs to help them navigate the digital patient portal. Some patients delegated responsibility for managing their health information in the portal to their CPs alone, whereas others sought to collaborate with their CPs in reading and understanding clinical notes:

My mother is now 82, and at that age, she’s not likely to engage with apps or registration issues. If anything, I managed it for her or we discussed it.

CP_0419

I can open this page and then we can read it together. Or I explain to him what my concerns were. But independently, no longer.

CP_0201

In contrast, a younger and more digitally literate patient reported that she was able to access her Open Notes independently and only sought assistance from her CP when she encountered issues:

Well, I would first read it [Open Notes] on my own because I only have my daughter as a relative. And she has her own problems at the moment, so I don’t really need her help unless I have issues. I’ll handle it myself first.

PAT_0402

The ability to access clinical notes enabled many CPs (6/10, 60%) to gain a deeper understanding of the illness and treatment of the family member with a severe mental health issue. This increased their confidence in providing effective care and managing the illness, allowing them to better support the treatment (such as preparing for medical appointments and adhering to medication plans):

It [Open Notes] significantly improved my understanding. I could better follow the treatment processes and medical decisions. This helped me support my mother better and make informed decisions.

CP_210

It [Open Notes] was sometimes difficult, especially when the reports were not positive. But it helped me be better prepared and respond quickly if something was wrong.

CP_0213

As previously indicated (see the *Changes in Contact and Relationship Dynamics* section), this heightened level of involvement and responsibility, in addition to the improved information flow, was partially attributable to more intensive contact with HCPs, as evidenced by the following quote:

Yes, the contact became more intense, and I felt more included in the treatment. It was a very positive effect that improved the collaboration.

CP_0210

A few CPs (3/10, 30%) reported that Open Notes enabled them to provide more effective support from a greater distance (eg, from another city or country). Furthermore, CPs observed that reading the clinical notes reduced the need to accompany patients to medical appointments, thereby enhancing autonomy for both CPs and patients:

This allowed me to monitor their health data and ensure they received the right care even when I couldn’t be with her. It gave me a sense of security to always be informed.

CP_0210

In addition to increased involvement, responsibility, and support, some CPs (3/10, 30%) also demonstrated a tendency to monitor or control patients through Open Notes. They compared the patient-reported information from medical appointments with the written information to assess the veracity of the reports and identify any potential omissions. This monitoring held the potential for conflict, but in one case, it also led to a more open and honest exchange between a patient and a CP regarding their inner motivations (eg, the withholding of information due to feelings of embarrassment, fear of disempowerment, or memory issues) and, thereby, enhanced mutual understanding:

It [Open Notes] allowed me to access all relevant information and better monitor my mother’s health. It helped us be better informed and respond more quickly to changes.

CP_0210

There was a moment of surprise when she didn’t mention something or had forgotten, but it was actually helpful because it led to a discussion where I could address it. She was honest, and we could discuss things in more detail or I could suggest she pay more attention to certain aspects. So, it wasn’t a bad thing; it facilitated further discussion.

CP_0416

#### Having Mixed Feelings

Both CPs (7/10, 70%) and patients (5/10, 50%) provided insights into their emotional perceptions of the Open Notes. Notably, both patients and CPs reported a similar range of emotional experiences, including both positive and negative feelings. Both groups reported feelings of emotional distress associated with reading about deteriorating health or lack of treatment success. One patient even described experiencing persistent worrying thoughts. Some patients (4/10, 40%) also expressed concern that their CPs might experience distress as a result of reading the notes:

Well, it’s a bit burdensome, I would say, maybe. When something new comes up and then the success doesn’t happen.

PAT_0402

It was a mix of relief and concern. Relief because I was informed, and concern when the information wasn’t positive. But overall, it helped me to be better prepared.

CP_0210

I am concerned that my relative may be emotionally distressed by reading the entries [Open Notes].

PAT_0207

Both CPs and patients noted that Open Notes provided a sense of security regarding the illness and its treatment. This sense of security was derived from 2 sources: first, the ability to access treatment information and, second, the knowledge that this information has been validated by experts:

No, for me it’s more like the lack of knowledge is stressful. When you have an informed status, you can handle it better.

CP_0211

Yes, actually good, because I know it comes from a competent source and not just from random internet readings where every third person says something different, and so on. So, for me, it’s reliable information. Definitely, knowing without having to worry about whether it’s true or not or maybe or something, so that’s more reassuring for me.

PAT_0402

One patient found it motivating and encouraging to read her HCP’s notes. In addition, this patient was particularly motivated by proxy access and the fact that her CP also read the notes, which led her to engage in more self-care. This particular finding suggests that access to clinical notes by CPs may also impact treatment outcomes or patient recovery on a personal level beyond the increased involvement of well-informed CPs in the patients’ health management:

But when you read the family’s comments, like, “Hey, you’ve been letting yourself go lately,” or “You seem unmotivated,” it motivates you. You realize they are right; there’s no reason to just hang around or whatever.

PAT_0411

In contrast, for other patients (2/10, 20%) and CPs (2/10, 20%), the clinical notes were less emotionally significant and were perceived more neutrally, as illustrated by the following quote:

You perceive it relatively neutrally. You don’t get super happy or deeply depressed.

PAT_0204

#### Looking Ahead: Suggestions and Feedback

Several patients and CPs provided feedback on potential modifications to the patient portal that could improve its usability. Typically, suggestions focused on modifying or enhancing existing features within the portal. For instance, 4 participants (n=3, 30% patients and n=1, 10% CPs) expressed a desire for a read receipt feature to confirm when HCPs had received and read their messages. In addition, 20% (2/10) of the patients proposed that they be notified when a response from their HCP had been submitted. This notification feature had already been incorporated into the system and could be enabled by the user, yet these patients were unaware of its availability:

Something like that, just like with emails where you can send a confirmation of receipt or read receipt, so you know it’s been received and opened. Sometimes, that’s all you need.

CP_0416

And if the HCP has written something, it would be nice to get an email notification so that I know there’s a message there. If it’s out of the ordinary and you don’t check it every day, you might not see it for a few days.

PAT_0204

Moreover, one patient expressed a desire for the portal’s features to be more appealing and engaging for older patients, with the goal of tailoring the portal’s design to the needs of this patient group (eg, by encouraging them to write comments). Both improved guidance and an optimized design were requested by the participants and could facilitate greater accessibility and appeal for the target audience:

A little guidance, maybe. Okay, that we start here, with a specific topic being set. I need to know, what should I write?

CP_0402

But the commenting function should be designed in a way that makes you want to use it, that makes you feel like speaking up.

PAT_0420

Other participants expressed a preference for integrating additional features into the patient portal beyond simply reading clinical documentation. In total, 10% (1/10) of the patients and 20% (2/10) of the CPs suggested enriching the patient portal with more psychoeducational information about the illness and integrating some type of psychoeducational lexicon or psychiatric frequently asked questions into the patient portal:

And I would certainly wish for a way to learn more about the illness, about behavioral strategies, options for the CP, but also for the patient. So you don’t have to Google and look for information elsewhere. If you’re already in the psychiatric system, maybe you could listen to more. Do you understand? That on this platform, on this level, you could already have specific questions answered.

CP_0201

In fact, one CP expanded the original scope of Open Notes by using the commenting feature to document important developments in her mother’s health. She used this primarily as a personal reminder while also indirectly facilitating transparency and understanding of progress for HCPs by posting it on the patient portal:

At the beginning, I would write down things that I noticed in my daily life with my mom, as a personal reminder. It was helpful to have these notes ready for the next appointment as preparatory points. I definitely find it useful for that.

CP_0409

## Discussion

### Synthesis of the Findings

The results highlight both opportunities and challenges associated with using Open Notes for older patients and their CPs. In addition, the key drivers of proxy access were identified, and their interdependencies were highlighted. Our results show that older patients and their (sometimes older) CPs must first gain (proxy) access to the patient portal to use and benefit from Open Notes. This requires sufficient digital literacy and mutual trust between patients and CPs. In our study, many older patients needed support from their CPs to navigate digital health services. Once access to open records was established, both parties reported feeling more informed about the illness and its treatment and more in touch with HCPs. These 2 factors led to increased health literacy, engagement, and involvement for both patients and CPs. In line with this, our results suggest that access to Open Notes enables CPs to better support patients in their (digital) health management. However, there was also evidence that CPs used Open Notes to control patients, which could lead to conflicts. Finally, recommendations for further developments and feedback emerged.

Our findings show that it is particularly valuable to allow patients, CPs, and HCPs to digitally engage with Open Notes via a comment function, allowing the stakeholders to directly communicate by sending asynchronous messages. This interactivity of the test environment (*PEPPPSY*) in which our study was conducted was frequently used and highly valued by patients and CPs. In particular, it appeared to contribute to the health literacy of patients and CPs by allowing them to ask questions about the content of the notes or the treatment in general. Communication via Open Notes is not a classic feature of Open Notes, nor is it simply a secure messaging function as the digital interaction is not separate from the Open Note itself. This demonstrates that Open Notes serve multiple purposes (such as providing information, facilitating contact, and offering reassurance) depending on the level of interactivity available [40].

### Ensuring Accessibility for Older Patients

As evidenced by previous research and observed in our study as well, older patients (and their CPs) predominantly use Open Notes as a memory aid, benefiting from this tool to prepare for or recap medical appointments. This provides both patients and CPs with an increased sense of security in their treatment processes. The positive effects of the implementation of Open Notes with a proxy access shown in this study—such as enhanced patient empowerment and engagement, increased CP involvement, and improved health management—align with those found in previous research [30,41,42]. Nevertheless, for these advantages to be fully realized, the initial challenge must be addressed: ensuring that older patients and their occasionally also older CPs have convenient access to the patient portal. This seems particularly relevant as older adults show interest in using patient portals yet the existence of numerous barriers hinders their ability to do so [19]. While CPs can indeed play a crucial role in compensating for the patients’ lack of digital health literacy—as observed in our study—it is equally important to encourage and enable older patients (with sufficient cognitive abilities) to independently access their health information. Older adults are often apprehensive or skeptical about digital health tools, so addressing these concerns is essential [43]. Furthermore, it is important to ensure that older patients are able to comprehend the content of the Open Notes. Consequently, the clinical documentation must be written in patient-friendly language, which is not always the case in clinical practice [6].

On the basis of the study results, it seems important to provide older patients and their CPs with a clear and detailed explanation of the available features (such as the commenting feature and the opt-in notification feature) before they use the patient portal and Open Notes. It is imperative that patient portal interfaces are designed in a manner that is accessible to all age groups and that the technical requirements are kept as user-friendly as possible. For instance, alternative methods of 2-factor authentication should be explored as requiring users to use 2 devices (eg, a phone and a computer) simultaneously can be overwhelming and frustrating and may, ultimately, result in older patients giving up on using the patient portal and Open Notes. At the same time, increased usability must be compatible with high-level data security requirements. Furthermore, the design of patient portal interfaces should adhere to fundamental age-specific design principles, including the use of appropriate fonts, color choices, and audio alternatives and the minimization of text entry requirements [44]. Providing users with the option to select either a *standard* or *older age–accessible* interface design when accessing the patient portal could prove advantageous as it would enable users to customize their experience to align with their specific requirements. Nevertheless, it seems unlikely that merely modifying the interface will be sufficient to significantly increase the adoption of patient portals and Open Notes among older patients, and therefore, a more comprehensive approach is needed.

First, older patients must be made aware of the availability of patient portals (and the possibility of setting up a proxy access) through comprehensive and targeted informational campaigns [45]. Second, older patients need to be encouraged to use patient portals through the aforementioned campaigns and, more importantly, through their general practitioners and other HCPs [46]. As highlighted in the interviews and supported by findings of other studies, human guidance is essential for older patients to use the full range of features available on patient portals [47]. This responsibility should not be borne solely by CPs, particularly given that not all older patients have access to a digitally literate CP [48].

In light of the ongoing digitalization of the health care system, it may be worthwhile to consider the introduction of an institutionalized role dedicated to this task. In the United States, the role of digital navigator is currently being investigated and defined [49]. Digital navigators are HCPs who have undergone specialized training in the area of digital mental health applications. They provide consultative assistance to health care providers and offer continuous guidance to patients in using these applications [50]. To date, this role has been primarily concerned with the use of digital health applications. However, in light of the increasing international adoption and promotion of EHRs and ORA, it may be beneficial to consider expanding the role of digital navigators to encompass these additional tools and consider the integration of artificial intelligence–assisted support—as proposed by Wunderlich et al [48] with the concept of digital case managers. Nevertheless, artificial intelligence solutions should not replace human guidance as one of the primary concerns of older patients is that digital services could potentially supplant personal contact with HCPs [43].

### Preserving the Autonomy of Older Patients

In addition to the aforementioned fundamental requirements for adapting patient portals to ensure accessibility for older adults, other aspects must be considered when involving CPs through proxy access. In the course of our study, the themes of trust, support, and control emerged as particularly salient, a finding that is also corroborated by other studies [30]. Despite the fact that CPs are only granted proxy access with the informed consent of the older patients, there is a risk that they may use Open Notes as a tool for control. This issue has significant ethical and practical implications [51]. Patients with dementia are especially reliant on the assistance of CPs in the management of their health [52], which also applies to the use of digital patient portals [53,54]. It could be argued that, particularly in cases in which patients are experiencing significant cognitive decline, such as with dementia, a certain level of control may be necessary and appropriate within the context of their care. Moreover, all participating older patients consented to the involvement of their relatives (and may revoke this consent at any time), thereby indicating their general assent to the sharing of information (and, thus, also to the potential for control). Nevertheless, this controlling behavior represents a substantial limitation of the patients’ autonomy and may potentially give rise to conflict in the relationship between patients and CPs. Furthermore, in accordance with the systemic *concept of a good reason*, it can be assumed that all behaviors, including the deliberate withholding of information by patients, are motivated by a good inner reason and represent a more or less constructive coping mechanism in the face of challenges and difficulties (eg, to avoid shame or to maintain independence) [55].

At this point, it is pertinent to re-examine whether these considerations are applicable to patients with age-related cognitive impairments. These reflections could likely be extended indefinitely, leading to a vicious circle. However, it is possible to diverge from this loop and conclude that it is essential to preserve the dignity of older patients (with and without dementia) while using ORA and Open Notes [56]. Therefore, it is crucial to consider how the experience of (controlling and) being controlled can develop into a trusting dependence on the support of CPs [57]. Caine et al [58] and Latulipe et al [30] suggest that patients should be informed precisely about which information CPs can access in the patient portal. It seems particularly important to provide patients with the option of fine-grained access settings, allowing them to decide which information should be shared and which should not [59]. Such fine-grained functionality was available in early versions of the Swedish national patient portal [3,60]. In addition, a *break-glass* access control protocol can be implemented whereby patients can define which information should be released in an emergency (eg, in the event of a significant deterioration in cognitive health or an unexpected hospitalization) [30,61]. Careful attention must be paid to defining the end points meticulously and distinctly (eg, establishing clear criteria for what constitutes significant cognitive decline).

### Implications

Older patients can benefit significantly from Open Notes with proxy access in their health care. However, to realize these benefits, older patients (and their CPs) must first be empowered to access the patient portal and understand clinical documentation. This requires adapting the design of patient portals to the needs of the older patient population and supporting the digital literacy of older patients through tailored individual and structural interventions. Enabling patients and CPs to interact with their HCPs through Open Notes seems to be a particularly important new feature. Many older patients rely on the support of CPs to manage their health care, especially when using digital health services. However, to ensure that older patients maintain their autonomy and dignity when using digital health services such as Open Notes, it is crucial to prevent these tools from becoming instruments of control for CPs. Older patients should be able to make granular decisions about what information they want to share with their CPs and what they want to keep private. For emergencies, a *break-glass* access protocol should be established in advance.

### Limitations

This study was based on a small number of participants, which limits the generalizability of the results. Furthermore, the group of participants was highly homogeneous with regard to the categories of race and migration status. To obtain generalizable results, larger studies with a more diverse selection of participants are required in the future. Similarly, older patients and their CPs represent a relatively specific participant group, which further limits the generalizability of the results. Younger patients and their CPs (eg, children or adolescents) may have differing user experiences and encounter completely different barriers and opportunities while using Open Notes. It is also necessary to consider the potential influence of social desirability bias. It can be assumed that older patients might have occasionally embellished their statements regarding their own digital literacy and the usability of the patient portal. For instance, a greater number of CPs than patients indicated that the patients experienced difficulties when using the portal. It is difficult to acknowledge one’s own shortcomings and limitations. Furthermore, it is particularly challenging to do so in the presence of others, such as CPs and interviewers, as this could lead to embarrassment and perceived loss of status. It should also be noted that the interviews were conducted via telephone. Although the participants were asked to find a quiet and secure place, it cannot be guaranteed that they were undisturbed throughout the interview. However, the telephone interviews allowed the older patients to remain in their homes (ie, they did not have to travel long distances) and have quick access to support from their CPs in case of difficulties (eg, technical problems or comprehension problems due to cognitive deficits). Thus, telephone interviews allowed older patients with physical or cognitive impairments, as well as CPs living in other cities or countries, to take part in the study and reduced barriers to study participation.

### Conclusions

Our study suggests that access to Open Notes can facilitate understanding and engagement between patients and their CPs and is associated with improved communication with HCPs. This may influence the dynamics of the triadic relationship among patients, CPs, and HCPs, with potential implications for power dynamics. In summary, no single patient portal can be expected to meet the needs of all patients—one size does not fit all. Individual solutions and adaptations of ORA are clearly needed to ensure acceptance and meaningful use by older patients and their HCPs.

## Appendix

Multimedia Appendix 1 Interview guides.

Multimedia Appendix 2 COREQ (Consolidated Criteria for Reporting Qualitative Research) checklist.

## Abbreviations

COREQ: Consolidated Criteria for Reporting Qualitative Research
CP: care partner
EHR: electronic health record
HCP: health care professional
ICD-10: International Classification of Diseases, 10th Revision
ORA: online record access
PEP.AGE: Piloting and evaluation of a participatory patient-accessible electronic health record for geriatric psychiatric patients and their care partners
PEPPPSY: Piloting and evaluation of a participatory patient-accessible electronic health record in Psychiatry and Somatics
