# Supplementary material for: Experiences of Older Mental Health Patients and Their Care Partners Using a Proxy Account to Access Open Notes: Qualitative Interview Study
Source: JMIR Aging. 2025 Feb 24;8:e66690. doi: 10.2196/66690 (PMC11894351; doi:10.2196/66690)
Supplement: Multimedia Appendix 1 [file aging_v8i1e66690_app1.pdf]

Interview guide PEP.AGE Care Partners

Thank you for agreeing to take part in the interview. I would like to ask you how you experienced the use of digitally shared documentation and what you may have found helpful or difficult. I am also interested in how you feel the use of digitally shared documentation has affected your treatment process and how you have experienced access to relatives.

First of all, in general terms, before I ask you a few more detailed questions: **How did you feel about participating in the study**?

**Topics**	**Open entry question**	**Concretizing demand**
Expectations in advance	What positive expectations did you have before using the shared documentation?<br><br>What negative expectations did you have before using the shared documentation?	What specific hopes or wishes did you have before using the shared documentation?<br><br>What specific worries, fears or anxieties did you have before using the shared documentation?
Dealing with digital technology	How did you manage to use the digital web application?<br><br>How did your relatives manage to deal with digital technology?	How did you find the log in? (challenging, easy, ...)<br><br>Did you need support with the setup and/or use?<br><br>Have you supported your relatives in using the technology?
Relatives	What experiences have you had with access to relatives?<br><br>How did you experience being able to view your relatives' personal documentation?	Have you benefited from having access to the clinical documentation?<br>□ What opportunities do you see for access to relatives?<br>□ e.g. support, reminder aid, increase adherence<br><br>Have you experienced difficulties and/or challenges?

	How did your relatives experience the fact that you have access to their clinical notes?	→ Privacy protection / What is shared?<br>→ Misunderstandings?<br>□ Feeling controlled<br><br>What feedback have you received from your relatives (the patient) regarding the use of shared communication?<br><br>What opportunities has the shared documentation created for your relatives (the patient)?<br><br>What difficulties and challenges did you experience for your relatives (the patient)?
Experiencing the process of reading the documentation	How did you feel when reading your practitioner's documentation?	Did you understand the content of the documentation? Was the documentation written in understandable language? If something was incomprehensible, how did you deal with it? (Follow-up questions via comment function, follow-up questions at the next appointment)<br><br>What thoughts and feelings did you experience while reading the clinical notes?<br><br>Did you experience any difficult or unpleasant feelings when reading the shared documentation?<br><br>How did you deal with these feelings?<br><br>Did you find any errors in the documentation? If so, how did you deal with them? (Comment function? Addressed at the next meeting?)

Contact to practitioners		Did you yourself feel the need to discuss entries with the practitioner?<br><br>Have you contacted the practitioners to talk about entries?<br><br>Has the contact between you and the practitioner changed? (e.g. being more involved in the treatment)
Education, transparency, adherence	How has the use of shared documentation influenced your understanding of your relative's illness?	If so, what do you think is the reason for this?
Self-manageme nt	Has the use of shared documentation helped you in dealing with your relative's illness? If yes, what exactly?	Do you have the impression that you can better support your relatives by reading the GD?<br>→ e.g. when preparing for visits to the doctor<br><br>Do you feel you have a better overview of your illness, treatment and progress to date?<br><br>Were you able to better remember or recall the content of the conversation by viewing it?
Comment function	Have you used the comment function?	If so, what did you use it for?<br>- Ask questions<br>- Self-organization, writing down notes for yourself<br>- Organizational matters<br>- Point out errors to the practitioner<br><br>Have you experienced any barriers or obstacles when using the comment function?<br><br>What opportunities do you see in using the comment function for treatment?

	How did the practitioner use the comment function?<br><br>Has the use of the comment function changed the way you communicate with the practitioners of your relatives?<br><br>What challenges have arisen from the use of the comment function?<br><br>In your opinion, has the use of the comment function had an impact on the treatment?	How promptly were your comments answered by the practitioners?<br><br>Has there been an exchange between you and the practitioners via the comment function?<br>Has communication become more direct or faster?<br>Has the frequency of contact increased?<br>Has contact become more interactive or more intensive?<br>Do you have the impression that you can reach your practitioners better through the comment function?<br>In your opinion, to what extent does the improved accessibility of your practitioner affect the quality of treatment?
Perspectives	Would you like to use the shared documentation on a permanent basis?	In your opinion, would something have to change for the successful implementation of shared documentation?
Conclusion	To what extent have your expectations - both positive and negative - regarding shared documentation been fulfilled?<br><br>Would you like to draw a personal conclusion, is there anything else you would like to say on the subject?	

PEP.AGE patient interview guide

Thank you for agreeing to take part in the interview. I would like to ask you how you experienced the use of digitally shared documentation and what you may have found helpful or difficult. I am also interested in how you think the use of digitally shared documentation has affected your treatment process and how you have experienced access of relatives (proxy access).

First of all, in general terms, before I ask you a few more detailed questions: **How did you feel about participating in the study**?

**Topics**	**Open entry question**	**Concretizing demand**
Expectations in advance	What positive expectations did you have before using the shared documentation?<br><br>What negative expectations did you have before using the shared documentation?	What specific hopes or wishes did you have before using the shared documentation?<br><br>What specific worries, fears or anxieties did you have before using the shared documentation?
Dealing with digital technology	How did you manage to use the digital web application?	How did you find the approach? (challenging, easy, ...)<br><br>Did you need support with the setup and/or use?
Experiencing the process of reading the documentation	How did you feel when reading your practitioner's documentation?	Did you understand the content of the documentation? Was the documentation written in understandable language? If something was incomprehensible, how did you deal with it? (Follow-up questions via comment function, follow-up questions at the next appointment)<br><br>What thoughts and feelings did you experience while reading the documentation?

		Did you experience any difficult or unpleasant feelings when reading the shared documentation?<br><br>How did you deal with these feelings?<br><br>Did you feel properly restored?<br><br>If you didn't feel properly restored, how did you deal with it?<br><br>Have you ever had the impression that your practitioner deliberately omitted something from the documentation, e.g. a sensitive or difficult topic?<br><br>Did you find any errors in the documentation? If so, how did you deal with them? (Comment function? Addressed at the next meeting?)
Contact to practitioners		Did you yourself feel the need to discuss entries with your practitioner?<br><br>Have you contacted your practitioners to talk about entries?
	Designing the contact<br><br><br><br><br><br><br>Decision making	Did the contact between you and your practitioner change? (e.g. more frequent, higher quality, longer/shorter)<br><br>In your opinion, has the relationship with your practitioner changed as a result of the shared documentation? If so, how?<br><br><br>Does the use of shared documentation make you feel more involved in treatment decisions than before?

Education, transparency, adherence	How has the use of shared documentation influenced your understanding of your condition?	If so, what do you think is the reason for this?
Self-manageme nt	Has the use of shared documentation helped you to manage your condition? If yes, what exactly?	Do you feel better prepared for doctors' visits by using shared documentation?<br><br>Do you feel you have a better overview of your illness, treatment and progress to date?<br><br>Were you able to better remember or recall the content of the conversation by viewing it?
Relatives	How did you experience that your relatives were also able to view the documentation?<br><br>What experiences have you had with the Proxy Access?<br><br>How did your relatives experience having access to your clinical notes?	Have you benefited from your relatives having access to the progress documentation? If so, how?<br>→ What opportunities do you see for access to relatives?<br>→ e.g. support, reminder aid, increase adherence<br><br>Have you experienced difficulties and/or challenges with access to relatives?<br>→ Privacy protection / What is shared?<br>→ Misunderstandings?<br>→ feeling controlled<br><br>What feedback have you received from your relatives regarding the use of shared communication?<br><br>What opportunities has the proxy access created for your relatives?<br><br>What difficulties and challenges did you experience for your relatives?

Comment function	Have you used the comment function?	If so, what did you use it for?<br>- Ask questions<br>- Self-organization, writing down notes for yourself<br>- Organizational matters<br>- Point out errors to the practitioner<br><br>Have you experienced any barriers or obstacles when using the comment function?<br><br>What opportunities do you see in using the comment function for treatment?
	How did your practitioner use the comment function?	How promptly were your comments processed by your practitioner?
	Has the way you communicate with your practitioner changed as a result of using the comment function?	Has there been an exchange between you and your practitioner via the comment function? Has communication become more direct or faster? Has the frequency of contact increased? Has the contact become more interactive or more intensive?
	Has the use of the comment function affected the contact with your practitioner?	Do you have the impression that you can reach your practitioners better through the comment function?<br>In your opinion, to what extent does the improved accessibility of your practitioner affect the quality of treatment?
	What challenges have arisen from the use of the comment function?	
	In your opinion, has the use of the comment function had an impact on your treatment?	

Perspectives	Would you like to use the shared documentation on a permanent basis?	In your opinion, would something have to change for the successful implementation of shared documentation?
Conclusion	To what extent have your expectations - both positive and negative - regarding joint documentation been fulfilled?<br><br>Would you like to draw a personal conclusion, is there anything else you would like to say on the subject?	
